# Why Does Tardive Dyskinesia Have Oro-facial Predominance? A Network Analysis

**DOI:** 10.1007/s10548-022-00931-y

**Published:** 2023-01-02

**Authors:** Krisztina Szalisznyó, David N. Silverstein

**Affiliations:** 1 Department of Medical Sciences, Psychiatry, Uppsala University, Uppsala University Hospital, 75185 Uppsala, Sweden; 2 Computational Sciences Department, Theoretical Neuroscience and Complex Systems Research Group, Wigner Research Centre for Physics, Budapest, 1121 Hungary; 3 Agora for Biosystems, Sigtuna Foundation, Sigtuna, Sweden

**Keywords:** Tardive dyskinesia, Orofacial dyskinesia, Cortico-striatal network, Adjacency matrix

## Abstract

Tardive dyskinesia is a involuntary hyperkinetic disorder which usually occurs in older patients after long-term treatment with antipsychotic drugs. These dyskinesias are mostly irreversible and are frequently expressed in the tongue, cheeks, mandible, perioral area and other regions of the face. In this theoretical study we asked the question, why does tardive dyskinesia often have orofacial predominance? What might be the underlying neural network structure which contributes to this propensity? Graph analysis of high-level cortico-striato-thalamo-cortical network structure suggests a connectivity bottleneck. The number of walks of different lengths from the substantia nigra pars reticulata (SNr) to other vertices, as well as the returning cycles are the lowest in the network, which may indicate a higher damage susceptibility of this node. Analysis was also performed on published data from a recent high resolution histological study on cortico-striato-thalamo-cortical networks in rodents. Finer network partitioning and adjacency matrices demonstrated that the SNr has a heterogeneous connectivity structure and the number of local walks from nodes neighboring orofacial neural representation is higher, indicating possible early compensatory escape routes. However, with more extensive SNr damage the larger circuit compensation might be limited. This area of inquiry is important for future research, because identifying key vulnerable structures may provide more targeted therapeutical interventions.

## Introduction

Tardive dyskinesia (TD) is an iatrogenic disorder and the underlying pathophysiology remains to be fully elucidated. TD patients can show volume reductions in subcortical regions, including within the basal ganglia (caudate nucleus, to a lesser extent in the putamen and minimally in the globus pallidus) and the thalamus (Sarró et al. 2013). The involvement of basal ganglia is in agreement with observations that early extrapyramidal side effects predict the onset of TD and this suggests important clinical implications (Tenback et al. 2009).

TD is most often caused by long-term use of drugs that block dopamine $D_{2}$ receptors. These $D_{2}$ antagonists may cause an increased sensitivity in the remaining $D_{2}$ receptors. This was proposed to decrease activity in the indirect pathway in the basal ganglia and presumably normal activity in the direct pathway (Ribot et al. 2019). Patients with TD are more likely to have their involuntary muscle movements confined to the oromandibular region with oro-facial-lingual stereotypies, compared with idiopathic patients (Tan and Jankovic 2000).

Gamma-aminobutyric acid (GABAergic) mediated striato-pallidal fibers play a crucial role in orofacial TD (Cools et al. 1989). The strength of some GABAergic synapses between the globus pallidus (GP) and the substantia nigrapars reticulata (SNr) increased after dopamine depletion, but not their short-term dynamics. This may cause an increase in the frequency and amplitude of spontaneous inhibitory synaptic events on SNr neurons. Synaptic proliferation can explain how dopamine depletion augments GABAergic transmission in the SNr and therefore contributes to the persistent dopamine hyper-sensitivity states (Faynveitz et al. 2019). During states with permanent reductions in dopamine, the effect has shown to be postsynaptic, in contrast to a short-term dopamine depletion which causes a presynaptic effect (Faynveitz et al. 2019).

A study performed on healthy monkeys showed that the non-selective dopamine agonist apomorphine activates both $D_{2}$ and to a much lesser extent $D_{1}$ receptors, which over time can cause orofacial dyskinesia and tic disorders that are a consequence of dopaminergic over-activity. These symptoms were related to reduced firing rates of SNr neurons and disinhibition of their targets (Nevet et al. 2004). A rodent study showed that the $D_{2}$ antagonist haloperidol decreases the number of cholinergic interneurons in the ventral lateral striatum and probably principal medium-spiny neurons (MSNs) as well, while producing tardive dyskinesia of the oromandibular region (Ribot et al. 2019).

Another rodent study demonstrated that the orofacial region of the SNr receives input directly from the striatal jaw region and sends output directly to the pontine and medullary premotor neurons, which in turn activates oro-facio-lingual motor neurons innervating the jaw, tongue, and facial muscles with an ipsilateral predominance (Inchul et al. 2005). Neuronal activation in central SNr can code the onset of entire rule-governed sequential patterns of grooming actions, not just elemental movements (Meyer-Luehmann et al. 2002).

### Hypothesis

**Fig. 1:**
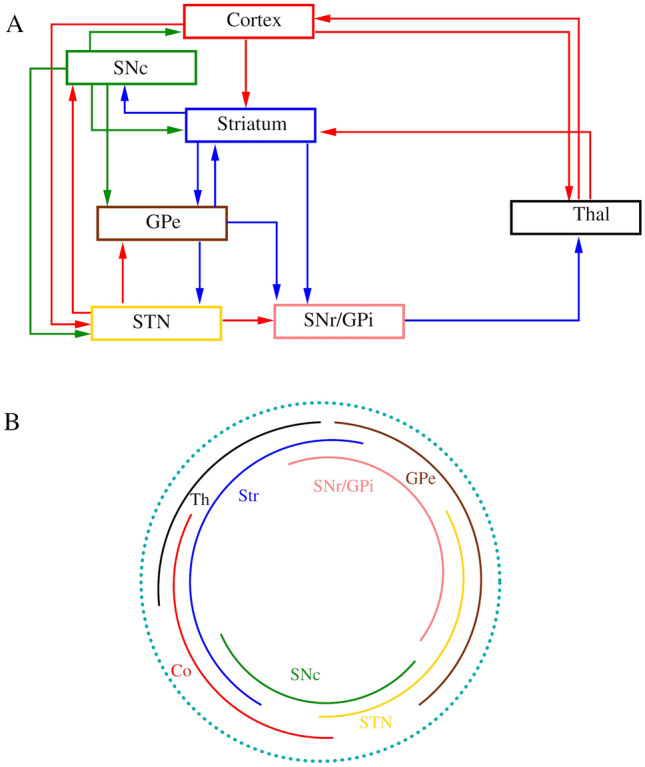
**A** Is a schematic representation of coarse connectivity in CSTC circuits. Blue represents GABAergic projections, red illustrates glutamatergic projections and green indicates dopaminergic projections. **B** Shows a circular-arc graph, with arc overlaps indicating connectivity, showing another view of the coarse CSTC network model

Given that TD can result from long-term use of dopaminergic drugs, we propose that analyses of anatomical intrinsic and extrinsic connectivity in relevant motor circuits can provide information on the orofacial susceptibility of topographically organized cortico-striato-thalamo-cortical (CSTC) networks during TD development (Simonyan 2019; Dybdal et al. 2013). A better understanding of how susceptible circuits such as SNr and globus pallidus externus (GPe) neurons as well as $D_{2}$-expressing MSNs change over time, has a potential to improve therapies while reducing TD symptoms. We assumed that adjacency relations in the connectivity matrices can pinpoint nodes with low and high connectivity potential, implicating vulnerabilities in these circuits (see Fig. 1).

## Results

**Fig. 2:**
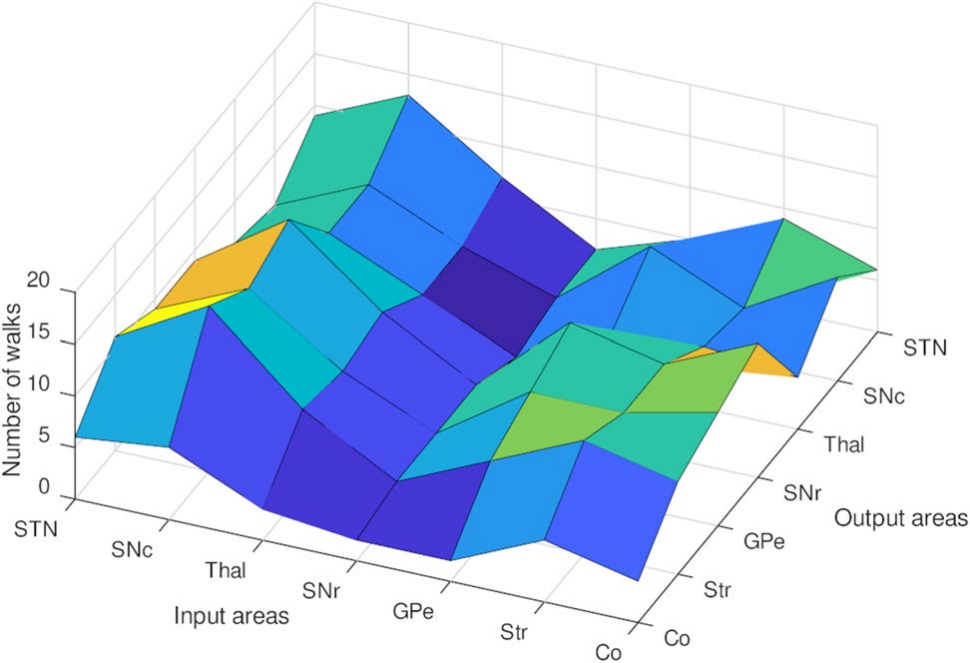
Number of walks from the given input areas to output areas in the coarse-grained CSTC network. Shown are the number of walks of length 4 ($k=$4)

**Fig. 3:**
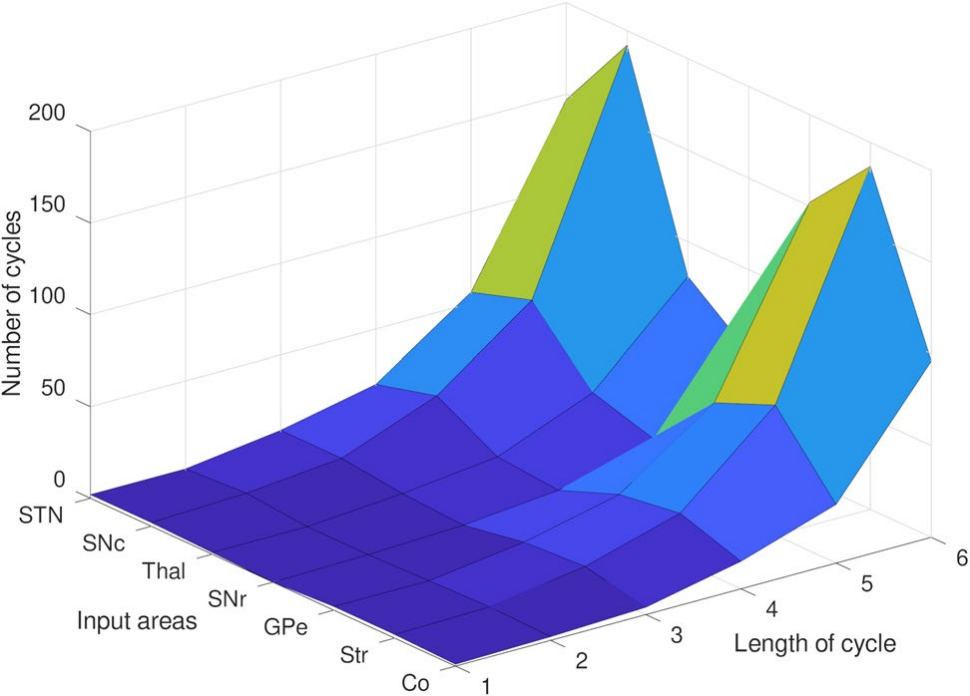
Number of cycles from and back to a given node in the coarse-grained CSTC network. Different cycle lengths were plotted. A trend showing the minimum number of cycles at the SNr is independent of the cycle length

By raising the coarse connectivity adjacency matrix to a power of 4 (for walks of length 4) the CSTC network shows a minimum number of walks from the node that represents SNr (Fig. 2). The diagonal (number of cycles) of this matrix also shows a minimum at the SNr node. When the lengths of walks are varied with powers of the adjacency matrix, the number of cycles at SNr remains consistently at a minimum (Fig. 3). These results imply that the SNr may be a connectivity bottleneck at the coarse connectivity scale. Damage to this structure, which has significant orofacial representations, may make it difficult to compensate by recruiting other parts of the network.

**Fig. 4:**
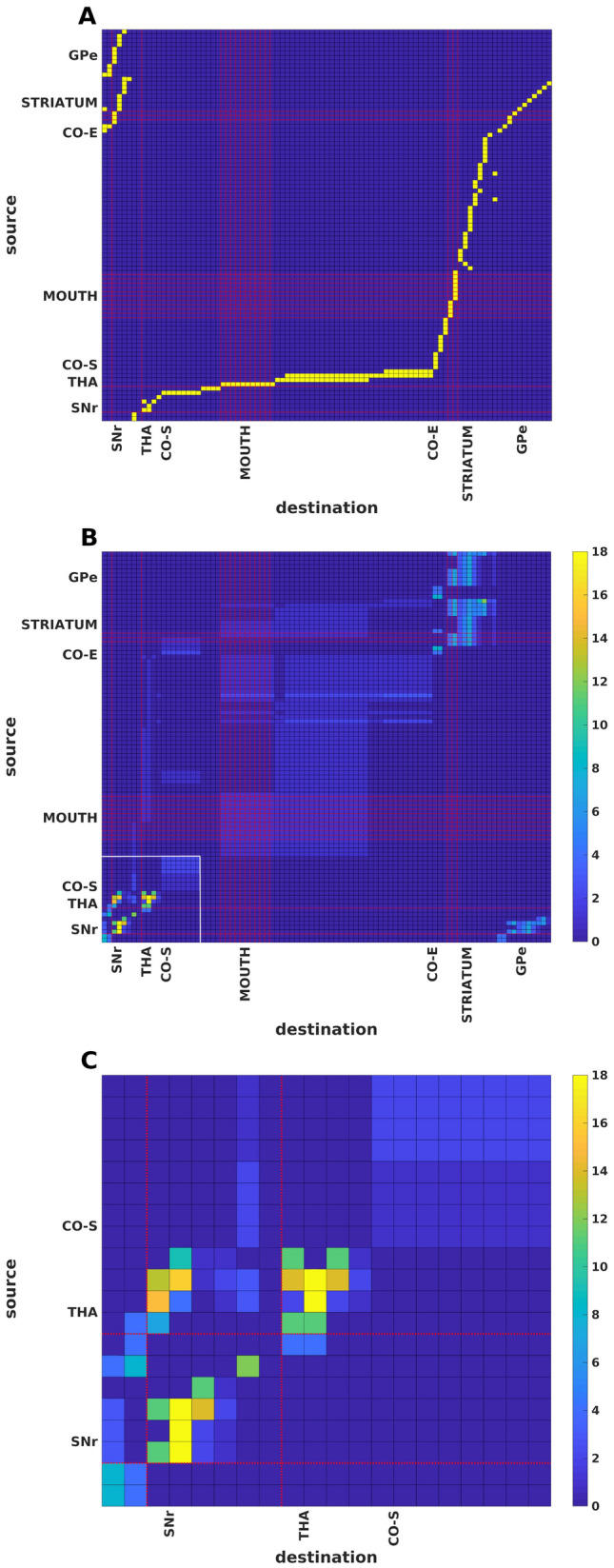
Adjacency matrices with fine resolution connectivity of CSTC networks, based on a recently published histological study (Foster et al. 2021). The red lines indicate brain regions with orofacial representation. CO-S and CO-E represents the start and end of cortical regions, respectively. THA denotes thalamus. **A** Adjacency matrix of the fine resolution connectivity, where yellow indicates connections. **B** The 4th power of the same adjacency matrix. The outlined white square is magnified in the next figure. **C** Magnification on the SNr, thalamus and part of cortex. The highest number of walks are in the SNr, bordering the areas with orofacial representation

A finer resolution connectivity matrix was constructed from a recently published histological investigation [data according to Fig. 4 from Foster et al. (2021)]. With further analysis, we found that the orofacial neural representation in the CSTC network follows a fine topography (Fig. 4). When the adjacency matrix is raised to a power, we found that the SNr has a more heterogeneous connectivity structure, incorporating subnodes with low but also high numbers of walks (Fig. 4B, C). The vertices which represent orofacial information are neighboring nodes with those which have high number of walks originating from them (Fig. 4C). Thus, a high resolution network analysis may indicate that local damage can be compensated for with alternative routes on a fine resolution scale, but in case of more extensive SNr injury there are limited compensatory circuits at a larger network level.

**Fig. 5:**
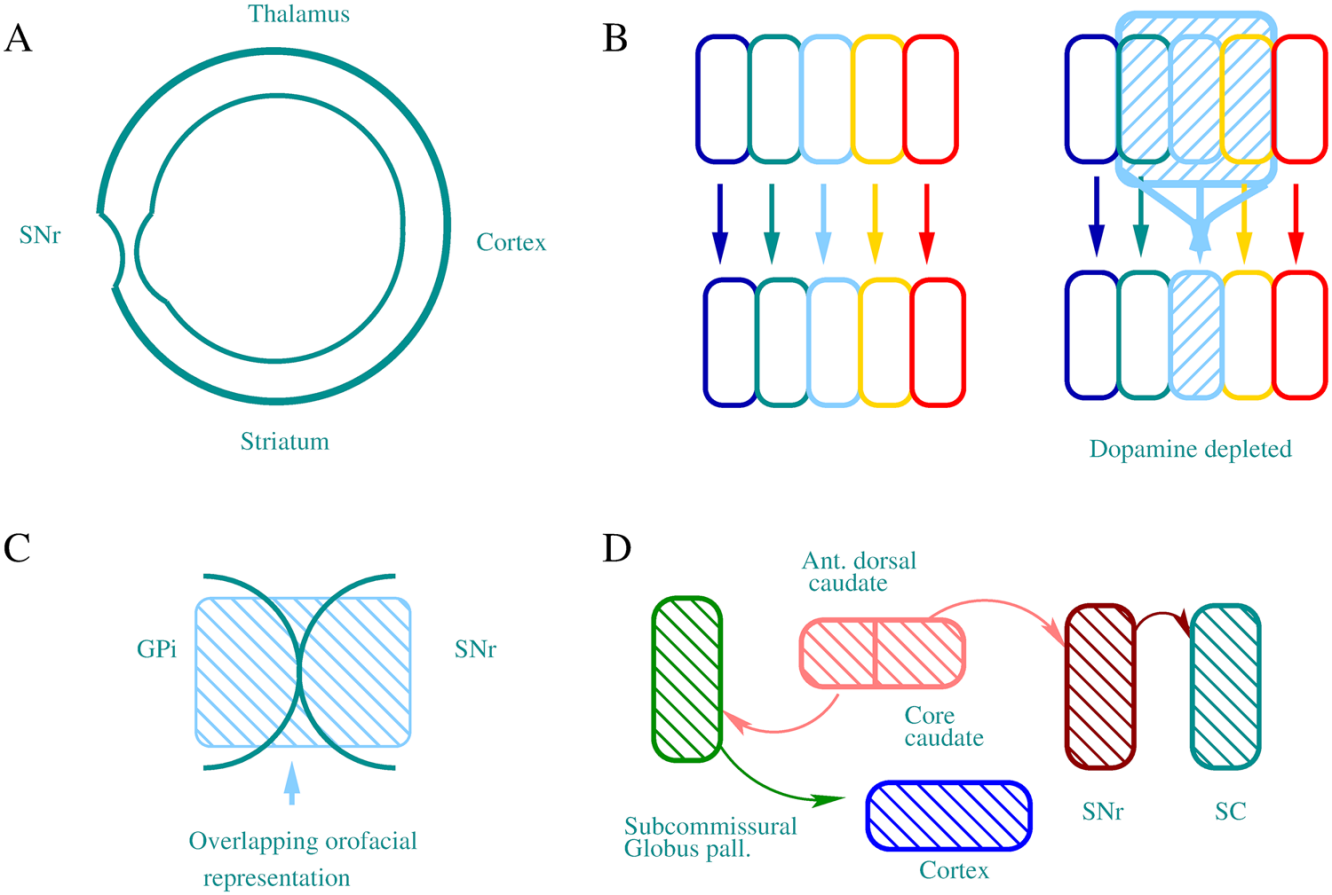
Schematic representation of some possible contributing factors in the development of TD etiology. **A** Connectivity bottleneck at the node level of SNr, which has high orofacial representation, as demonstrated in this study. **B** With states of dopamine depletion (e.g. antipsychotic medication induced), downstream neurons in the cortico-striato-thalamo-cortical circuits become responsive to a wider range of inputs. **C** The orofacial representation, that extends from GPi to the adjacent region of SNr. The orofacial representation is divided between posteroventral portions of GPi and adjacent regions of SNr (DeLong et al. 1983). **D** Experimental evidence from animal studies suggest that signals from SNr to superior colliculus (SC), (Gunne et al. 1988; Deniau et al. 1996), as well as the pathway from caudate via subcommissural globus pallidus may play role in oral dyskinesia development (Cools et al. 1989)

## Methods

We created adjacency matrices from coarse and fine resolution CSTC closed loop connectivity data. The coarse connectivity adjacency matrix was generated from the directed graph shown in Fig. 1. The fine resolution connectivity data came from Foster et al., (2021), (Fig. 4). For a graph *G* with vertex set *V*(*G*) of size *n*, its adjacency matrix is an $n \times n$ (square) matrix, where $A(G)=[a_{i,j}]$ describes the adjacency relations between all pairs of vertices in the matrix, where its element is 1 when *i* is adjacent to *j* and 0 otherwise (Fig. 1, Matrix 1). The entries along the diagonal of the adjacency matrix give the number of walks (cycles) from a given vertex to itself. For any graph *G*, the kth power of its adjacency matrix $A_{(v,u)}$ contains elements which count the number of walks of length *k* from the vertex *u* to the vertex *v*.

Connectivity analysis was performed using Octave (octave.org) version 5.2.0 with Ubuntu 20.04 Linux.

**Matrix 1:**
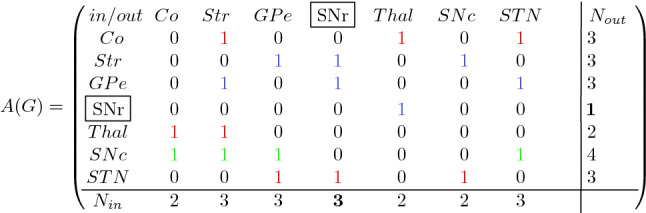
Adjacency matrix of coarse connectivity in the CSTC circuits (From Fig. 1). Blue represents GABAergic projections, red indicates glutamatergic projections and green represents dopaminergic projections. The abbreviations Co, Str, GPe, SNr, Thal, SNc, STN represent cortex, striatum, globus pallidus externus, substantia nigra pars reticulata, thalamus, substantia nigra pars compacta and subthalamic nucleus, respectively. SNr denotes in this matrix the combination of SNr and GPi. *N*_*in*_ and *N*_*in*_ indicate the number of incoming and outgoing edges for every node.

## Limitations

Emerging studies have begun to show higher connectivities of the Globus pallidus in primates and this was not included in the coarse connectivity analysis. This study used a simplified view of coarse human connectivity and a recent rodent study for more detailed CSTC topography, which may translate only partially to humans. The structure of the number of cycles and walks with different lengths and the number of synaptic connections possible via the direct and indirect pathways might be directly related to specific vulnerabilities in the CSTC pathologies. Further study should investigate in detail the number of walks and cycles in the direct and indirect pathways. Single cell properties can also contribute to altered firing patterns in SNr neurons (Cáceres-Chávez et al. 2018), which was not a focus in this work. A recent rodent study demonstrated that certain cortical oro-brachial subnetworks directly project to the SNr (Foster et al. 2021). Future work should investigate this and other larger network contributions in TD development.

## Discussion

Long-term use of antipsychotics can lead to dopaminergic hypersensitization on MSNs and interneurons in the striatum. This might result in an imbalance between the direct and indirect pathways and an abnormal output to the sensori­motor cortex, contributing to abnormal movements and symptoms of TD (Yu et al. 2021). Persistent exposure to $D_{2}$ antagonists reduce firing rates in $D_{2}$ expressing MSNs, which may also cause MSN and downstream degradation of synapses along the indirect pathway. It may also be that reduced GPe inhibition of the thalamic reticular nucleus, which is GABAergic and inhibits other encapsulated thalamic nuclei, could potentially cause some spurious motor activity. Patients with tardive oromandibular dystonia are more likely to have their symptoms confined to the oromandibular region, compared with idiopathic patients (Inchul et al. 2005).

In a rat model of L-DOPA induced dyskinesia (LID), increased blood-brain barrier permeability was demonstrated in the lateral striatum, in SNr and in globus pallidus internus (GPi) (Cenci 2014). In LID, increase in synchronized afferent activity was found, which drives SNr oscillations and is associated with abnormal involuntary movements, suggesting the potential use of desynchronizing drugs for managing this condition (Meissner et al. 2006). There is a low risk to patients of dyskinesia induced from $D_{2}$-like receptor agonists or to drug-naïve animals with nigrostriatal lesions. In contrast, $D_{2}$ receptor antagonists can produce tardive dyskinesia in patients (Murer and Moratalla 2011).

The striatonigral direct pathway displays a greater convergence of striatal inputs than the more parallel striatopallidal indirect pathway. Direct and indirect pathways originating from the same striatal domain converge onto the same postsynaptic SNr neurons (Foster et al. 2021). Electrophysiological studies demonstrated a lower degree of informational convergence in GPe and a higher degree in SNr. The greater specificity of the indirect pathway is likely to have a functional significance. In monkeys with states of dopamine depletion, GPe neurons become responsive to a wider range of striatal inputs (Foster et al. 2021) (Fig. 5B). TD symptoms are known to be funneled via the GABAergic striatonigral pathway to the SNr and then to the intermediate layers of the superior colliculus (Cools et al. 1989) (Fig. 5D). Reductions in firing rates and regularity in SNr neurons was observed after dopamine depletion, which can be associated with homeostatic mechanisms.

Pars reticulata cells are in extensive communication with each other, with morphology characterized by extensive local axon collaterals. This confirms earlier extracellular electrophysiological observations indicating strong inhibitory interconnections between these neurons (Hajós and Greenfield 1994). This inhibition contributes to dynamic circuit organization for activating and executing ongoing grooming sequences. Because neurons in the SNr are triggered at sequence onset of syntactic grooming chains, they may be especially tuned to the initiation of sequential motor patterns (Meyer-Luehmann et al. 2002).

In primates, the dorsal one-third (centrolateral portions) of SNr carries the orofacial representation as a continuation of the same regions of the GPi (Simonyan 2019). Somatotopic maps are mainly within the GPi, except for the orofacial representation, that extends from GPi to the adjacent region of SNr. This indicates that the two output nuclei, GPi and SNr may form a single, conjoint representation of pre-frontal and motor cortical territory (Shipp 2017; Hoover and Strick 1999), thus the orofacial representation is divided between posteroventral portions of GPi and adjacent regions of SNr (DeLong et al. 1983). The apparently greater proportion of SNr neurons related to orofacial than to limb movement might account for the prevalence of orolingual movements in this disorder (Fig. 5C).

The localization of orofacial neurons in the centrolateral region of the SNr and the weak projections from the face area of the motor cortex to this region of the SNr suggests a somatotopic organization of this nucleus (DeLong et al. 1983). A previous study identified a distinctive topography of abnormal postural and motor responses evoked by transient focal inhibition of the monkey SNr (Dybdal et al. 2013). The “onion-like” distribution of striatal inputs is similar to that observed in the distribution of nigral efferent neurons. The nigral lamination underlies formation of specific input-output channels of processing. The orofacial sensorimotor channel, which receives afferents from orofacial cortical areas, projects to subsets of collicular and thalamo cortical networks that contribute to head and orofacial movements (Deniau et al. 1996) (Fig. 5D). It should be noted that SNr may be primarily associated with ocular and orofacial movements, while GPi is associated with movements of the limbs (Nevet et al. 2004). Further studies should dissect the finer details of network topography related to TD symptoms, taking into consideration larger network contributions as well.

## Conclusion

In conclusion, we found a connectivity bottleneck in the coarse topological properties of the CSTC functional network (Fig. 5A). However, analysis based on finer resolution studies revealed inhomogeneity in the SNr, where certain parts of this node has higher numbers of walks and compensatory routes, consistent with the findings that there are extensive local axon collaterals between the SNr neurons indicating strong inhibitory interconnections (Hajós and Greenfield 1994). In CSTC circuits there are parallel channels, each of which constitutes serial focal connections from node to node through the loops. However, consistent with our results, some stages utilize more diffuse connectivity, in which each microchannel connects with all others typically at the level of to GPi/SNr (Shipp 2017). Clinical studies on Parkinson’s disease (PD) patients has shown that deep brain stimulation (DBS) of the SNr but not of the Subthalamic nucleus (STN) was better at controlling anticipatory postural adjustments and gait control, and DBS has been shown to be feasible in PD patients with dyskinesia as well (Cury et al. 2022). The implications of our result are that specifically focused therapeutic improvements towards the SNr, perhaps through the STN (Lagière et al. 2020) could help in the plastic reorganization of the network to decrease the induction of repetitive oro-facial movements.

Our results are consistent with previous experimental findings, which have shown that an apparently greater proportion of SNr neurons are related to orofacial than to limb movement. This might partially account for the prevalence of orolingual movements in TD (DeLong et al. 1983).

## Data Availability

Data for the coarse-grained analysis of CSTC network connectivity was from referenced studies described in Introductionsection and summarized in Fig. 1, with the generated adjacency matrix shown in Matrix 1. The fine-grained CSTC network connectivity analysis was based on (Foster et al. 2021), Fig. 4. The generated fine-grained adjacency matrix is available from the corresponding author on request.
